# Randomized controlled clinical trial evaluating the efficacy of hyperbaric oxygen therapy in facilitating the healing of chronic foot ulcers in diabetic patients: the study protocol

**DOI:** 10.1186/s13063-020-04757-6

**Published:** 2020-09-29

**Authors:** Jocefábia Reika Alves Lopes, Mariza D’Agostino Dias, João Antonio Correa, Maria Alice Bragagnolo Batalha, Luanda Karla Dantas Guerra

**Affiliations:** 1 Department of Vascular Surgery, Centro Universitário Saúde ABC, Santo André, São Paulo, Brazil; 2 Department of Medical Sciences, University of São Paulo, São Paulo, Brazil; 3 Department of Plastic Surgery, Municipal Hospital of Imperatriz, Imperatriz, Maranhão Brazil; 4 Department of Vascular Surgery, Municipal Hospital of Imperatriz, Imperatriz, Maranhão Brazil

**Keywords:** Diabetes mellitus, Diabetic foot, Hyperbaric oxygenation, Wound healing, Randomized controlled trials

## Abstract

**Background:**

Diabetic limb ulcers are highly prevalent and contribute to a significant increase in cost for the treatment of these patients in health services. However, healing of these wounds is a major health problem and may even lead to amputation. The primary aim of the current study is to evaluate the efficacy of hyperbaric oxygen therapy (HBOT) in facilitating the healing of diabetic foot ulcers, in addition to secondarily evaluating whether it reduces the number of amputations and improves the quality of life in these patients.

**Methods:**

A non-blind randomized clinical study will be conducted in the city of Imperatriz, Maranhão state, Brazil, from 2019 to 2020, in diabetic patients with chronic foot ulcers (classified as Wagner grades 2, 3 and 4, persisting for more than 1 month). The outpatient follow-up for diabetic foot patients will be done at the Unified Health System, with a sample size of 120 patients (the randomization allocation will be 1:1, being 60 patients for each arm). Half of the patients will receive standard treatment, i.e. dressings, debridement, antibiotics and load relief, along with HBOT (HBOT group), and the other half will receive only standard treatment (control group). The patients of the HBOT group will be evaluated upon admission, after 10, 20, 30 and 35 HBOT sessions, and after 6 months and 1 year. The patients of the control group will also be evaluated at equivalent periods (upon admission, after 2, 4, 6 and 7 weeks, 6 months and 1 year). The SF-36 quality of life questionnaire will be filled upon admission and after 3 months of follow-up in both groups. The primary and secondary endpoints will be assessed with 1 year of follow-up.

**Discussion:**

Diabetic foot ulcers are a highly prevalent complication of diabetes with serious consequences. A study to assess the efficacy of HBOT in healing the ulcers and reducing the rate of amputations in diabetic patients is justified, which will eventually aid in the development of guidelines for treating these ulcers.

**Trial registration:**

Registration number RBR-7bd3xy. Registered on 17 July 2019—Retrospectively registered.

## Background

Globally, more than 400 million adults suffer from diabetes, and the treatment of diabetic foot ulcers is considered as an important public health issue [1]. According to the Ministry of Health of Brazil, the number of diabetic patients in Brazil increased by 61.8% between 2006 and 2016, and the prevalence increased from 5.5 to 8.9%, with more prevalence in women (9.9%) than in men (7.8%). Approximately one in every twenty diabetic patients develops foot ulcers in the first year, and approximately 10% of the patients with ulcers evolve to amputation in this period [2, 3].

Unhealed and infected wounds in diabetic foot cause damage in both tissues and bones, which leads to amputations in 85% of these individuals, and approximately 60% mortality has been reported after amputation during 5 years of follow-up [4–6].

Chronic changes that occur in the feet of diabetic patients, such as peripheral arterial disease, neuropathy with loss of protective sensitivity, deformities and decreased mobility of the feet, are the main challenges in the prevention and treatment of diabetic foot ulcers [7, 8]. The treatment of diabetes and diabetic foot ulcers requires multiple approaches, which involve optimization of glycaemic control, wound care, treatment of infections, load relief and revascularization in ischemic cases [2, 9]. Unfortunately, even with optimal care, the rate of complete healing of the wounds stays approximately 60% per year [2, 10]. Hyperbaric oxygen therapy (HBOT) has been used as an adjuvant therapy for patients with refractory ulcers [11, 12].

Normal wound healing occurs through the ordered and overlapping stages of haemostasis, inflammation, proliferation and tissue remodelling, involving complex molecular and cellular interactions within the wound microenvironment [13]. Regardless of the aetiology of the wound, an adequate condition of the vasculature, including both macrocirculation and microcirculation, is critical for healing. Insufficient perfusion impairs angiogenesis, collagen deposition and epithelialization and may lead to sustained inflammation [14].

Among the advanced therapeutic interventions for wounds, HBOT has the unique ability to improve tissue hypoxia, reduce pathological inflammation and mitigate ischemia-reperfusion injury [15].

Evidence on the effectiveness of HBOT in healing diabetic foot ulcers is variable. Some researchers have reported greater effectiveness when HBOT is compared to sham treatment or placebo [2, 16–19], but others found no differences [7, 20]. HBOT has also been reported to promote the resolution of infection and reduce the likelihood of amputation by some authors [17], but others have shown no benefit [7, 21].

A systematic review of randomized clinical trial data recently published by the Cochrane Collaboration [22] reported a significant improvement in short-term (6 weeks) wound healing, but no statistically significant difference was found in wound healing rates for long-term amputation and major or minor amputation favouring HBOT, thus, suggesting the need for further randomized studies to clarify these doubts.

### Objectives

#### Primary objective

The primary objective is to assess whether HBOT along with the standard treatment of chronic diabetic foot ulcers is more effective in wound healing than the standard treatment alone.

#### Secondary objective

The secondary objective is to assess whether HBOT, along with the standard treatment of chronic diabetic foot ulcers, reduces the number of major (transtibial and transfemoral) and minor amputations (toes and forefoot) and improves the quality of life of these patients, as compared to the standard treatment alone.

### Hypothesis

Adding adjunctive treatment of HBOT to the standard treatment of diabetic foot ulcers, i.e. dressings, debridement, antibiotics and load relief, can improve wound healing and reduce major amputations, improving the quality of life of these patients.

## Methodology

### Study design

This is a parallel, two-arm, non-blinded, randomized controlled trial in the single centre.

### Study site

The study will be conducted in the city of Imperatriz, Maranhão, in diabetic patients with chronic foot ulcers (classified as grades 2, 3 and 4 of Wagner [23] and persisting for more than 1 month). The outpatient follow-up for diabetic foot patients will be done at the SUS (Unified Health System - a public health service for the entire Brazilian population), with HBOT sessions being held in the CicatrizAR Clinic.

### Sample and study period

The sample will consist of diabetic patients with chronic foot ulcers treated with standard methods, i.e. dressings, debridement, antibiotics and load relief (control group), and to the other group will be added HBOT (HBOT group). The study will be conducted from 2019 to 2020.

The sample size has been calculated based on the formula for comparing two independent groups according to qualitative variables [24], with 95% confidence interval and 80% power. A wound healing rate of 90% was considered achievable for the case group at 1 year, which we expected to be at least 20% higher than in the control group (absolute difference, i.e. no more than a wound healing rate of 70% was expected in the control group). Thus, the total sample size was estimated as 60 patients per group (120 in total).

### Trial status

Registration number RBR-7bd3xy. Registered 17 July 2019. The first recruitment was held on 4 July 2019 and the last recruitment is foreseen on 31 August 2020.

### Eligibility criteria

The inclusion criteria of the study are adult patients (age > 18 years); stable clinical presentation; diabetes type 1 and 2; Wagner grades 2, 3, and 4 foot ulcers; ulcers persisting for more than 1 month without cure; authorization for the study; and patients of the SUS (Unified Health System).

Exclusion criteria of the study are failure to meet one of the inclusion criteria, patients with macroangiopathy (two distal pulses absent) and patients with contraindications for HBOT, absolute or relative, patients on bleomycin chemotherapy, with chronic obstructive pulmonary disease, previous spontaneous pneumothorax, chronic sinusitis, chronic otitis media, unstable angina, severe congestive heart failure, claustrophobia, severe dementia, depression and history of seizures.

### Data collection

**Fig. 1:**
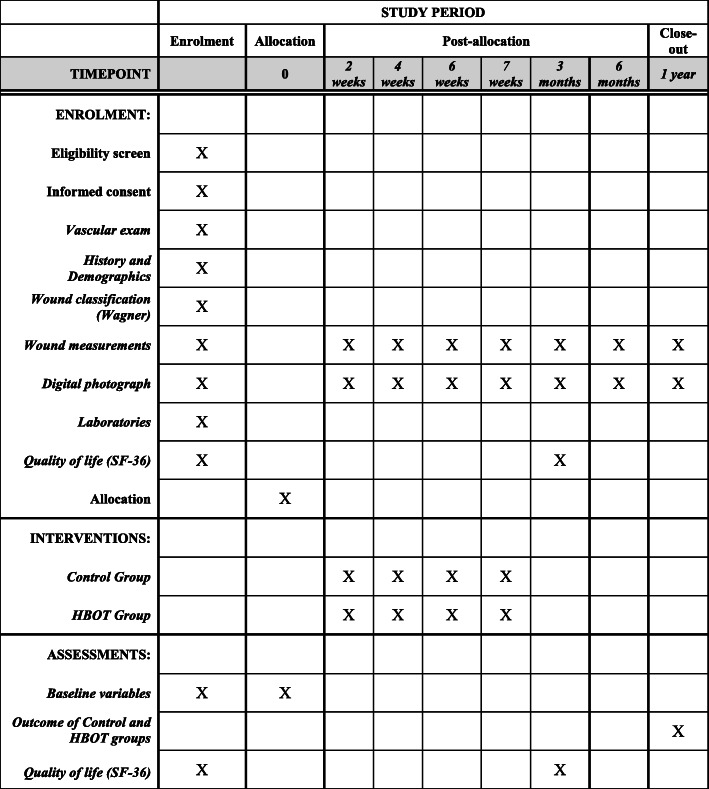
The schedule of enrolment, interventions and assessments

The data collection will be performed in the diabetic foot outpatient clinic of the SUS and in the CicatrizAR clinic, using a standardized questionnaire provided in the Supporting Information, as well as with photographic recording of the ulcers, according to the timeline described in Fig. 1.

### Randomization

This is a parallel, two-arm, non-blinded, randomized controlled trial in which an randomization will be performed by the research coordinator, who will carry out by simple 1:1 draw (for each pair of patients, one participant will select the number 1 or 2 in the draw, which will correspond to the group to be allocated, while the second patient will be allocated to the other group.). The randomization will not be done by computer-generated random numbers. Patients will be selected at the SUS diabetic foot clinic, where they regularly monitor the lesions on their feet. They will be evaluated according to the inclusion criteria of our research and, if they meet the criteria, they will be invited to participate in the research. The researchers explain how the study will be carried out and after 1 week, if the patient is interested in participating in the study, the research coordinator collects the signature of the patient’s consent form that found in the support information for this article, as well as performs the randomization of patients to the research groups.

### Interventions

The patients of the HBOT group will be evaluated upon admission, after 10, 20, 30 and 35 HBOT sessions and after 6 months and 1 year, and the control group will also be assessed at equivalent periods (upon admission, after 2, 4, 6 and 7 weeks, 6 months and 1 year) to clinically evaluate the ulcers and perform specific measures using the software ImageJ, developed at the National Institutes of Health (NIH, Bethesda, Maryland). HBOT sessions will be held 1 week after randomization. The sessions will be supervised by doctors trained in hyperbaric medicine by the Brazilian Society of Hyperbaric Medicine (SBMH). The HBOT sessions will be conducted 5 days a week, being carried out with patients in a multiplace chamber at 2.5 atm absolute (ATA) and 100% O_2_, with 10 min for compression and decompression, effective sessions of 90 min, with intervals of 5 min for each 30 min of treatment. The dressings will be done in both groups with Exufiber foam Ag® (*Molnlycke*, Gotemburgo, Suécia) or Mepilex® (*Molnlycke*, Gotemburgo, Suécia), which will be chosen according to the characteristics of wounds, with silver dressings for the infected injuries and a foam with polyurethane protective layer for wounds in which granulation tissue has formed. Secondary dressings will be changed daily and the primary dressings (coverings—Exufiber foam Ag or Mepilex) will be changed every 3–5 days, as needed and in both groups. The progression of the wounds and specific treatment, such as appropriate bandage, antibiotic therapy or need of some surgical intervention, will be evaluated on a weekly basis. In both groups, a vascular surgeon will monitor the patients, and who will assess the need for surgical procedures (debridement and amputations), in addition to appropriate antibiotic therapy for each case. With regard to load relief, patients in both groups will wear orthopaedic Baruk shoes.

The SF-36 quality of life questionnaire will be filled upon admission and after 3 months of follow-up in both the groups. Laboratory examinations, such as a haemogram, erythrocyte sedimentation rate, levels of C-reactive protein, creatinine, fasting blood glucose and glycosylated haemoglobin will also be estimated only on admission.

### Primary outcome

Wound healing will be assessed by evaluating the diameter of the lesions by specific software and periods, as described in the interventions. The wound healing will be achieved when there is no more skin lesion on the skin and the primary endpoint will be a binary result. The assessor of the primary outcome will be blinded.

### Secondary outcome

Amputation rates and the reduction of lesions that do not heal will be assessed, with an evaluation of statistical significance between the groups. The evaluation of the domains of the SF-36 quality of life questionnaire will also be done in both groups. The data from this questionnaire upon admission and after 3 months of follow-up will be compared.

### Data analysis

The collected data will be stored in a Microsoft Excel 2016 spreadsheet format. After checking for errors and inconsistencies, descriptive summaries will be provided by treatment arm, including absolute and relative frequencies and measures of central tendency and variability. We will do regular monitoring, with active search by address and phone to avoid sample loss. We will use the intention-to-treat in the analysis. Blinding will be done for the researcher who will evaluate the final data and for the statistician.

Logistic regression models will be used to assess associations between the categorical variables. Both relative effects (odds ratios) and absolute effects (risk differences) will be presented with corresponding confidence intervals. Student’s *t* test or a similar non-parametric method will be used for the analysis of continuous variables. All examinations will be performed at 5% significance in the IBM SPSS® program, Version 24.0, 2016 (IBM, Armonk, NY, USA).

### Ethical aspects

This study will be based on the principles of Resolution 466/12 of the National Health Council that regulates the research involving human subjects. The patients involved shall be duly informed and clarified about the importance and purpose of the study; if patients accept to participate, they will sign an informed consent form (see supporting information). The non-participation in the study or waiver, privacy, reliability and anonymity of the participants will be guaranteed.

The study was initiated after approval by the Research Ethics Committee of the ABC University. It is registered with the Brazilian Registry of Clinical Trials (ReBEC) under the number RBR-7bd3xy.

### Adverse events

This study is being conducted in accordance with the guidelines of the Brazilian Society of Hyperbaric Medicine (SBMH) and Undersea & Hyperbaric Medical Society (UHMS), and if adverse events occur (such as dizziness, seizure,7 pneumothorax, pneumomediastinum, nauseas, middle ear barotrauma, seizure, ear pain, confinement anxiety, hypoglycemic event and shortness of breath), these will be conducted according to these guidelines, as well as be documented and published later.

## Discussion

The current study has been designed to compare the existing standard treatment for chronic foot ulcers of diabetic patients, i.e. dressings, debridement, antibiotics, load relief and their combination with HBOT. Since we do not believe in comparing the two groups with pseudotherapy, it will not be performed on any patient as described by O’Reill et al. and Londahl et al. [7, 25]. Subpressurising or pressurinsing as a placebo would alter the normal physiology of patients’ blood gases, which does not occur in standard treatment. Therefore, we believe in the power of evidence that can be provided with this proposed study model.

## Supplementary information

**Additional file 1.** Research Questionnaire.

**Additional file 2.** Opinion of the Research Ethics Committee.

**Additional file 3.** Informed Consent Form.

## Data Availability

Data and materials will be available as soon as they are collected. The datasets used and/or analysed during the current study available from the corresponding author on reasonable request. The results of the study will be published in a scientific journal, as well as via Plataforma Brasil and ReBEC.

## Abbreviations

HBOT: Hyperbaric oxygen therapy
OR: Odds ratio
SUS: Unified Health System
