# Supplementary material for: Randomized controlled clinical trial evaluating the efficacy of hyperbaric oxygen therapy in facilitating the healing of chronic foot ulcers in diabetic patients: the study protocol
Source: Trials. 2020 Sep 29;21:816. doi: 10.1186/s13063-020-04757-6 (PMC7526398; doi:10.1186/s13063-020-04757-6)
Supplement: Supplementary file 1 — Additional file 1. Research Questionnaire. [file 13063_2020_4757_MOESM1_ESM.docx]

## RESEARCH QUESTIONNAIRE

**DATA COLLECTION**

**IDENTIFICATION:**

NAME: ______________________________________________________________

AGE: _____ Date of birth:______________ SEX: M( ) F( ) Tel: __________________

Height: __________________ Weight: _____________ BMI: ________

SMOKER:  YES ( ) NO ( )

DIABETES:  TYPE 1 ( ) TYPE 2 ( )

DURATION OF DIABETES IN YEARS:__________________

MEDICATIONS:  ORAL ANTI-DIABETIC DRUG ( )

INSULIN ( )

COMORBIDITY:  HYPERTENSION ( )

HEART DISEASE ( )

KIDNEY DISEASE ( )

RETINOPATHY ( )

Prior STROKE ( )

Prior Heart attack ( )

Prior AMPUTATION ( )

DIAMETER OF ULCER IN cm: WOUND < 3 cm ( )

WOUND ≥ 3 cm ( )

DURATION OF ULCER IN MONTHS: _____________________________

CLASSIFICATION OF ULCERS: WAGNER GRADE 2 ( )

WAGNER GRADE 3 ( )

WAGNER GRADE 4 ( )

LABORATORY EXAMS: HAEMOGLOBIN: ________________

LEUCOGRAM: _________________

ESR: ____________

CRP: ____________

FASTING GLUCOSE: ____________

GLYCOSYLATED HAEMOGLOBIN:_______

CREATININE: ____

##

## Diameter of the ULCER in cm (FOLLOW-UP)

ON ADMISSION: ___________________________

AFTER 10 SESSIONS / 2 WEEKS: ____________________________

AFTER 20 SESSIONS / 4 WEEKS: ____________________________

AFTER 30 SESSIONS / 6 WEEKS: ____________________________

AFTER 30 SESSIONS / 7 WEEKS: ____________________________

AFTER COMPLETING 6 MONTHS: __________________________

## AFTER COMPLETING 1 year: __________________________
