# Supplementary material for: Randomized controlled clinical trial evaluating the efficacy of hyperbaric oxygen therapy in facilitating the healing of chronic foot ulcers in diabetic patients: the study protocol
Source: Trials. 2020 Sep 29;21:816. doi: 10.1186/s13063-020-04757-6 (PMC7526398; doi:10.1186/s13063-020-04757-6)
Supplement: Supplementary file 2 — Additional file 2. Opinion of the Research Ethics Committee. [file 13063_2020_4757_MOESM2_ESM.docx]

**CONSOLIDATED OPINION of the Research Ethics Committee**

**Title of the Study:** Does hyperbaric oxygen therapy facilitate healing of chronic foot ulcers in diabetics?

Investigator: Jocefabia Reika Alves Lopes

Version: 2

CAAE: 10809819.9.0000.0082

Proponent Institution: Fundação do ABC – FMABC

Primary Sponsor: Own Funding

OPINION DATA

Opinion number: 3.345.550

Project presentation:

Globally, more than 400 million adults suffer from diabetes, and the treatment of diabetic ulcers of the lower limbs is considered as an important public health issue (1). According to the Ministry of Health of Brazil, the number of Brazilians with diabetes increased by 61.8% between 2006 and 2016, and the prevalence increased from 5.5% to 8.9%, with higher prevalence in women (9.9%) than in men (7.8%). Approximately one in every twenty diabetic patients develops foot ulcers in the first year, and approximately 10% of the patients with ulcers require amputation in this period (2, 3). Unhealed and infected wounds in diabetic foot can cause damage to both tissues and bones, which leads to amputations in 85% of these individuals, and 60% mortality has been reported after amputation during the five year-follow-up (4-6). Chronic changes that occur in the feet of diabetic patients, such as peripheral arterial disease, neuropathy with loss of protective sensitivity, deformities, and decreased mobility of the feet are the main challenges in the prevention and treatment of diabetic foot ulcers (7). The treatment of diabetes and diabetic foot ulcers requires multiple approaches, which involve optimisation of glycaemic control, wound care, treatment of infections, load relief and revascularisation in ischemic cases (2, 8). Unfortunately, even with optimal care, the rate of complete healing of the wounds stays below 60% per year (2, 9). Hyperbaric oxygen therapy (HBOT) has been used as an adjuvant therapy for patients with refractory ulcers (10, 11).

Normal wound healing occurs through the ordered and overlapping stages of haemostasis, inflammation, proliferation, and tissue remodelling, involving complex molecular and cellular interactions within the wound microenvironment (12). Regardless of the aetiology of the wound, an adequate condition of the vasculature, including both macrocirculation and microcirculation, is critical for healing (13).

Among the advanced therapeutic interventions for wounds, HBOT has the unique ability to improve tissue hypoxia, reduce pathological inflammation, and mitigate ischemia-reperfusion injury. The majority of the conditions in which HBOT has been used are known to have a few successful alternative treatments, but the morbidity and mortality associated with the failure of these alternative treatments is significant (14).

Evidence on the effectiveness of HBOT in healing diabetic foot ulcers is variable. Some researchers have reported greater effectiveness when HBOT is compared to sham treatment or placebo (2, 15-18), but others found no differences (7, 19). HBOT has also been reported to promote the resolution of infection and reduce the likelihood of amputation by some authors (16), but others have shown no benefit (7, 20).

A systematic review of randomised clinical trial data recently published by the Cochrane Collaboration (21) reported a significant improvement in short-term (6 weeks) wound healing, but no statistically significant difference was found in wound healing rates for long-term amputation and major or minor amputation favouring HBOT, thus, suggesting the need for further randomised studies to clarify these doubts.

**Study Objectives:**

 Primary objective:

To evaluate whether HBOT assists in the healing of chronic foot wounds and reduces the risk of major amputations in diabetic patients.

 Secondary objectives:

- To evaluate the healing rates of chronic diabetic foot ulcers that will be treated with and without HBOT; - To estimate the reduction rates of size of chronic diabetic foot ulcers that will be treated with and without HBOT; - To measure the reduction rates of major amputations (forefoot, transtibial, and transfemoral) in patients treated with and without HBOT; - To analyse the quality of life in patients with and without HBOT.

**Risk and Benefit Assessment:**

Risks:

Complications inherent to HBOT, which will be minimised following all national and international protocols governing the therapy (Protocols of the Brazilian Society of Hyperbaric Medicine and protocols of Undersea and Medical Society)

Benefits:

Optimisation of chronic wound healing in the feet of diabetic patients and reduction in the amputation rates in these patients.

**Comments and Considerations on the Study:**

This is a randomised clinical trial that aims at assessing whether HBOT facilitates the healing of chronic foot ulcers of diabetic patients, since there are no studies of this scope conducted in Brazil, and external studies still report conflicting results. Moreover, the latest Cochrane systematic review published in 2015 considered the need for further randomised studies to assist in the definition of this answer.

**Considerations on the presentations of Compulsory Documents:**

The following mandatory documents have been submitted for consideration by this committee:

Pending Response Letter

Project

Informed Consent Form

**Recommendations:**

Semester and Final reports are to be submitted to the Research Ethics Committee in the form of a notification.

**Conclusions or Pending Issues and List of Inadequacies:**

Project Approved.

**Final Considerations at Research Ethics Committee's discretion:**

The Research Ethics Committee of the ABC Faculty of Medicine, according to the attributions defined in CNS Resolution No. 466 of 2012 and CNS Operational Norm No. 001 of 2013, expresses the approval of the proposed research project.

This opinion is based on the following related documents:

| Document Type | Archive | Delivered | Author | Status |
| --- | --- | --- | --- | --- |
| Basic information  on the project | PB_INFORMAÇÕES_BÁSICAS_DO_PROJETO_1300065.pdf | 26/04/2019  15:03:41 |  | Accepted |
| Other | CartaRespostaPendencias.pdf | 26/04/2019  15:01:25 | Jocefabia Reika  Alves Lopes | Accepted |
| Other | CartaRespostaPendencias.docx | 26/04/2019  15:00:35 | Jocefabia Reika  Alves Lopes | Accepted |
| Detailed Project/Investigator’s brochure | ProjetoOHB.pdf | 26/04/2019  14:59:31 | Jocefabia Reika  Alves Lopes | Accepted |
| Detailed Project/Investigator’s brochure | ProjetoOHB.docx | 26/04/2019  14:56:48 | Jocefabia Reika  Alves Lopes | Accepted |
| ICF / Terms of Consent /  Justification of  Absence | TCLE.docx | 26/04/2019  14:56:19 | Jocefabia Reika  Alves Lopes | Accepted |
| Other | DeclaracaoFinanciamento.pdf | 29/03/2019  16:42:55 | Jocefabia Reika  Alves Lopes | Accepted |
| Other | DeclaracaoFinanciamento.docx | 29/03/2019  16:41:36 | Jocefabia Reika  Alves Lopes | Accepted |
| Other | Carta_de_Submissao_ao_CEP.docx | 29/03/2019  16:40:05 | Jocefabia Reika  Alves Lopes | Accepted |
| Cover Sheet | FolhadeRosto.pdf | 22/03/2019  19:22:03 | Jocefabia Reika  Alves Lopes | Accepted |
| Other | CartaCEP.pdf | 21/02/2019  13:32:06 | Jocefabia Reika  Alves Lopes | Accepted |
| Other | Carta_de_anuencia.pdf | 21/02/2019  05:26:00 | Jocefabia Reika  Alves Lopes | Accepted |
| Other | Declaracao_de_liberacao_de_local.pdf | 21/02/2019  05:25:22 | Jocefabia Reika  Alves Lopes | Accepted |
| Other | Carta_de_aceite_de_orientacao.pdf | 21/02/2019  05:20:43 | Jocefabia Reika  Alves Lopes | Accepted |

**Opinion Status:**

Approved

**Needs CONEP Appraisal:**

No

SANTO ANDRE, 24 de Maio de 2019

_________________________________

**Signed by**

**JUVENCIO JOSÉ DUAILIBE FURTADO**

**(Coordinator)**
