# Supplementary material for: Randomized controlled clinical trial evaluating the efficacy of hyperbaric oxygen therapy in facilitating the healing of chronic foot ulcers in diabetic patients: the study protocol
Source: Trials. 2020 Sep 29;21:816. doi: 10.1186/s13063-020-04757-6 (PMC7526398; doi:10.1186/s13063-020-04757-6)
Supplement: Supplementary file 3 — Additional file 3. Informed Consent Form. [file 13063_2020_4757_MOESM3_ESM.docx]

**Informed Consent Form**

**Does Hyperbaric Oxygen Therapy Facilitate Healing of Chronic Wounds in Diabetic Feet?**

You are being invited to participate in a research study designed to evaluate the use of hyperbaric oxygen therapy in the healing of chronic foot wounds in diabetic patients, as well as in the reduction of major amputations.

You may count on the assistance of the investigator, if necessary, at every stage of participation.

The benefits you should expect from your participation will be better healing of your wound.

The risks inherent in Hyperbaric Oxygen Therapy are related to increased oxygen support under pressure, which favors seizures, and is also intolerable for patients with severe retaining lung disease and severe heart disease; and is subjected to a hyperbaric environment (under pressure above atmospheric), where there is a need for equalization of pressure in the middle ear, and can not have infections of the upper respiratory tract and in the ear, as well as previous pulmonary lesions that under pressure. may favor the onset of pneumothorax. Because the chamber environment is closed, some patients may not adapt very well. Another important factor is that it is an oxygen-rich environment, with no metallic material being allowed into the chamber, as the clothes are 100% cotton and provided by the clinic for all patients. Thus, patients who have contraindications for hyperbaric oxygen therapy will not be able to participate in this research, such as: bleomycin chemotherapy - may potentiate the side effects of this drug, as well as the aforementioned changes such as chronic obstructive pulmonary disease, previous - spontaneous pneumothorax, sinusitis. chronic, chronic otitis media, unstable angina, severe congestive heart failure, claustrophobia, severe dementia, depression and a history of seizures.

You will be provided information on each stage of the study. You may refuse to continue participating in the study at any time or withdraw your consent without suffering any penalty, loss, or detriment to the continuity of your medical treatment.

Your identity and the information obtained by your participation will be viewed only by those responsible for the study, and the dissemination of the aforementioned information will only be made between professional scholars of the subject. You will not be identified in any publication that may result from this study.

You will be compensated for any and all expenses you may incur due to your participation in this study as well as for any and all damages that may occur for the same reason, as resources are guaranteed for these expenses.

If you have questions and/or concerns about your rights as a participant in this study or are dissatisfied with the way the study is being performed, you may contact the ABC School of Medicine’s Research Ethics Committee (CEP) at Avenida Príncipe de Gales, 821 – 1° Andar – Prédio CEPES, Santo André-SP; or by telephone at (11) 49935453. Our office hours are Monday through Friday from 8:30 AM to 4:00 PM. The ethics committee is responsible for monitoring and evaluating the ethical aspects of all research involving human beings to ensure the protection, dignity, rights, safety, and well-being of the research participants.

I declare that after proper clarification by the researcher and having understood what was explained, I consent to participate in this research project at the Cicatrizar clinic.

_______________________________________________

Chief Researcher

Dr. Jocefábia Reika Alves Lopes

FMABC and SBACV Registered Vascular Surgeon

SBACV and CBR Registered Vascular Sonographer

Council Number: CRM-MA 5312

ADDRESS: Rua Alagoas, 296, Bairro Jussara, Imperatriz-MA.

CONTACT INFO: (99) 35244622 / (99) 30727131

_______________________________________________

Research participant

Identity document number:

Sex: Male ( ) Female ( )

Date of Birth:

Imperatriz, MA, ______/_____/______
